# Effect of Soy Isoflavone on Hot Flushes, Endometrial Thickness, and Breast Clinical as well as Sonographic Features

**Published:** 2018-03

**Authors:** Marziyeh VAHID DASTJERDI, Bita ESLAMI, Maryam ALSADAT SHARIFI, Ashraf MOINI, Leila BAYANI, Hoda MOHAMMAD KHANI, Sadaf ALIPOUR

**Affiliations:** 1. Dept. of Obstetrics and Gynecology, Arash Women’s Hospital, Tehran University of Medical Sciences, Tehran, Iran; 2. Breast Disease Research Center (BDRC), Tehran University of Medical Sciences, Tehran, Iran; 3. Dept. of Radiology, Arash Women’s Hospital, Tehran University of Medical Sciences, Tehran, Iran; 4. Dept. of Obstetrics and Gynecology, Vali-e-Asr Hospital, Tehran University of Medical Sciences, Tehran, Iran; 5. Dept. of Surgery, Arash Women’s Hospital, Tehran University of Medical Sciences, Tehran, Iran

**Keywords:** Isoflavone, Hot flushes, Breast tissue, Endometrial thickness

## Abstract

**Background:**

Phytoestrogens treatment to relieve hot flushes in menopausal women was considered recently. However, the actual effectiveness and safety are not clear.

**Methods:**

Randomized clinical trial (IRCT#20100706004329N5) was performed in 204 patients who complained of hot flushes in Arash Women’s Hospital, Tehran, Iran from 2013–2015. The first group received 50 mg isoflavone (group A) once daily and the second group received placebo (group B) in the same regimen. Patients were evaluated for breast examination (BE) and breast sonography (BS) as well as vaginal sonography at initial presentation and at 6th and 12th week follow-ups. Patients were educated to record frequency and severity of hot flushes.

**Results:**

Group A experienced less hot flushes attack (6 vs 9 patients with 5< attacks in 6 wk (*P*= 0.05), 7 Vs 13 patients with 5< in 12 wk (*P*=0.01)) which was also less severe (8 vs 12 patients with severe symptoms in 6 wk (0.04) and 3 Vs 10 patients with severe symptoms in 12 wk (0.001). Isoflavone had no effect on neither breast density nor nodularity (in 6 wk, *P*=0.50 and 0.80, respectively and in 12 wk, *P*=0.32 and 0.43) and neither breast tenderness nor nipple discharge (in 6 wk, *P*=0.40 and 0.34 respectively and in 12 wk, *P*= 0.31 and 0.26). There were no significant differences in endometrial thickness in 6 and 12wk.

**Conclusion:**

Effects of isoflavone on frequency and severity of hot flushes in perimenopausal women is observed. Some clinical and ultrasonographic benign non-significant changes of the breast and endometrium are noted after isoflavone intake.

## Introduction

Decreasing levels of estrogen would trigger uncomfortable symptoms of hot flushes, night sweats, sleep disturbances, and vaginal dryness during women’s menopausal transition (1). Hot flushes have been reported as the most bother-some symptom of menopause by many women, which may significantly affect the quality of life (2). Although, hormone replacement therapy (HRT) is an established treatment for these symptoms and may effectively reduce vasomotor symptoms associated with decreased estrogen levels during menopause, but it has been refused by women (3) because of recent concerns about increased risks of stroke and venous thromboembolism (VTE), breast cancer and heart attack. As a result, a dramatic increase in enthusiasm for herbal treatment was seen which was later supported by evidence-based clinical trials (4–7). Phytoestrogens (PEs) are nonsteroidal plant compounds with similar chemical structure to estradiol, and these compounds appear to demonstrate an estrogenic or anti-estrogenic effect depending on the circulating estrogen level (8). The two main categories of PEs are Isoflavones and Lignans; soybeans are rich in Isoflavones, and Lignans can be found in whole grains, legumes, fruits, vegetables and flaxseed (9). Asian women would experience much less frequently vasomotor symptoms than women in America or Europe thus an increasing attention has been paid to different dietary habit (10) as Asian diet is rich in PEs (11). Hence, a large amount of research has been devoted to finding if PEs are effective in the treatment of menopausal symptoms, but so far results have been inconclusive (12).

PEs effect on breast tissue is one of the subjects of interest for organ-specific effects of these compounds. Low breast cancer rates in Japan has been hypothesized to be associated with the diet than to genetic protective factors; as traditional Japanese diet is consist of the high daily consumption of PEs (200 mg/die) (13).

The majority of studies which investigated the actions of PEs had used a pure unconjugated form but available products on market typically contain mixtures of various PEs with poorly defined chemical composition (14). Thus, little attention has been paid to the effects of mixtures of these compounds.

In this study, we followed three goals. The first was to evaluate the efficacy of isoflavone as one of the most frequently used forms of PEs in relieving menopausal symptoms. The second was to investigate the effects of PEs on the endometrium, and the last one was to reveal the effect of PEs on breast examination and ultrasonographic features.

## Materials and Methods

This randomized clinical study (IRCT#20100706004329N5) was performed on patients referring to Gynecology And Obstetrics Outpatient Clinic of Arash Women’s Hospital, Tehran University of Medical Sciences, Tehran, Iran from 2013–2015.

The Institutional Review Boards of our department approved study protocol and all patients gave informed consent.

Women over 40 yr of age with body mass index in the range of 20–35Kg/m^2^ who complained of hot flushes were chosen for our goals. Women with previous history of cancer, diabetes mellitus or renal, hepatic, and heart failure or abnormal uterine bleeding, myoma, ovarian cyst, Poly Cystic Ovarian Syndrome (PCOS), endometriosis, hormone therapy during the last three months, consumption of drugs interacting with intestinal absorption, a known history of breast disease or the detection of breast mass or nodules in breast examination were excluded. In addition, patients who had a suspicious history of probable sensitivity to soy products, disability or known history of drug or alcohol consumption, cigarette smoking, and caffeine consumption were excluded.

The patients were divided into 2 groups based on their arrival order to clinic; the first group received soy extracts as 50 mg isoflavone in form of a tablet, one before lunch and one before dinner (soy menopause tablets – nature made) and the second group were given placebo resembling the active drug in size and shape in the same manner. Both groups had the regimen for 12 wk. At the first visit, patients underwent breast examination (BE) and breast sonography (BS). Breast examination was performed by two board-certified general surgeon oriented with the aim of the study. Prior to the study, breast tenderness and nodularity in BE were scored each from 1–4 shown in Table 1. BS was performed by single board-certified radiologist and breast compositions were categorized according to American College of Radiology (ACR) 5^th^ edition 2013 as follows: a) Homogenous background echotexture-fat, b) Homogenous background echotexture-fibroglandular, c) Heterogenous background echotexture. The hot flushes were assessed by a chief resident of gynecology and were categorized in mild, moderate and severe. Endometrial thickness was measured at one-third of the endometrial cavity, with the calipers being positioned between the hyperechogenic endometrial area and the surrounding hypoechogenic halo. A double-layer measurement was recorded. Each patient was followed for a period of 12 wk, with evaluations being made after 6 wk, and then at 12 wk. At each checkpoint, the patients were asked to record the daily number of hot flushes and describe its severity. They also underwent trans-vaginal ultrasonography (TVS) for the measurement of endometrial thickness. BE and BS has been made as well. Moreover, patients were educated to examine breast tissue themselves and return to the clinic as if they find any abnormality.

**Table 1:** Breast examination categorization

***Tenderness***	***0***	***1***	***2***	***3***
Nodalarity	No	One Localized	Multiple Localized	Diffuse
Breast Thickenings	No	One Localized	Multiple Localized	Bilateral
Nipple Discharge	No	One duct	Multiple Ducts	Bilateral

The data were analyzed using SPSS software version 22 (Chicago, IL, USA). The continuous variables are reported as means ± SD, and number and percentage report categorical variables. Normality for continuous variables was determined by the Shapiro-Wilk test, which revealed that the continuous variables showed normal distributions (*P*>0.05). The unpaired t-test was used for comparison of variables between groups. For statistical evaluation of categorical variables, we used the Pearson chi-square test and Fisher’s exact test as appropriate. *P* values of <0.05 were accepted as significant.

## Results

**Fig. 1:**
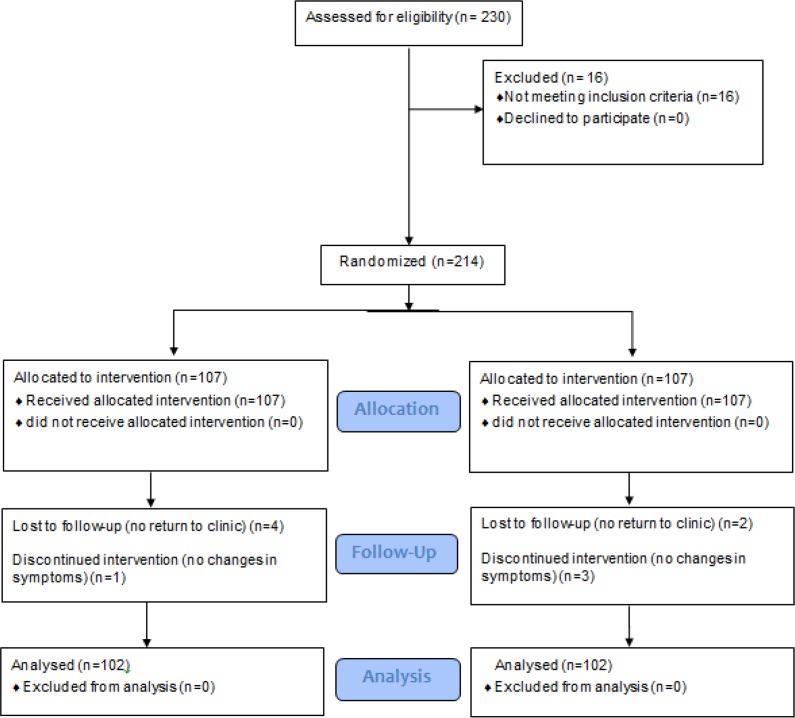
CONSORT Flow diagram for patient recruitment

During the follow-up period, one patient in isoflavone treated group (A) and 3 in the placebo group (B) quit the study as they claimed no changes were happening in their symptoms (Fig. 1). Thus 204 patients were examined, 102 patients in each group. Patients’ age was ranged from 41 to 59 yr. There were no significant differences in patients’ demographics between two groups (Table 2).

**Table 2:** Basal characteristics of study population

***Variables***	***Group A (n =102)***	***Group B (n =102)***	***P-value***
Age (yr)	51.5±1.5	51.8±1.7	0.18
BMI (kg/m^2^)	24.7±2.3	25.1±2.1	0.20
Age of menopause (yr)	47.6±3.1	47.3±2.4	0.44
Duration of menopause (yr)	2.7±1.9	2.9±1.7	0.43

Data are expressed as mean ± standard deviation. *P*-value refers to student *t*-test

There was a significant difference seen between two groups regarding number and severity of hot flushes in different follow-up periods. As group A experienced less hot flushes attack in each follow-up which was also less severe (Table 3). Endometrial thickness was evaluated in each follow-up in both groups but there were no results stating significant differences (Table 3).

**Table 3:** Clinical symptoms and sonographic evaluation of both group with 102 patients inT_0_ (baseline), T_1_ (after 6 wk), T_2_ (after 12 wk)

***Variable***	***T_0_***		***T_1_***		***T_2_***	
	**Group A**	**Group B**	**Group A**	**Group B**	**Group A**	**Group B**
**Hot flashes (n)**						
- *2>*	27	29	39	33	45	30
- *2–5*	65	62	57	60	50	59
- *5<*	10	11	6	9	7	13
***P*-value**	0.30		**0.05**		**0.01**	
**Hot flashes severity**						
- *Mild*	32	29	41	30	48	28
- *Moderate*	58	60	53	60	51	64
- *Severe*	12	13	8	12	3	10
***P*-value**	0.43		**0.04**		**0.001**	
**Endometrial Thickness (mm)**	3.4±1.9	3.3±1.95	3.2±1.78	3.2±1.6	2.9±1.4	3.00±1.5
***P*-value**	0.23		0.36		0.73	
**Breast sonography**						
- *A*	91	93	95	96	96	98
- *B*	11	9	7	6	6	4
- *C*	0	0	0	0	0	0
***P*-value**	0.47		0.58		0.63	

Data are expressed as number. *P*-values refer to chi-square test, Fisher's exact test or student *t*-test when appropriate

The isoflavone had no effect on breast tissue evaluated by BE, as both groups did not show any statistically significant difference in both breast density and nodularity nor breast tenderness and discharge (Table 4).

**Table 4:** Breast examination results in T_0_ (baseline), T_1_ (after 6 wk), T_2_ (after 12 wk)

	***T_0_***		***T_1_***		***T_2_***	
	**Group A**	**Group B**	**Group A**	**Group B**	**Group A**	**Group B**
**Breast Thickenings**						
Normal	86	89	90	89	92	91
One localized	9	7	7	10	6	8
Multiple Localized	7	6	5	3	4	3
Bilateral	0	0	0	0	0	0
***P* value**	0.68		0.50		0.32	
**Breast Nodularity**						
No Nodule	95	92	95	89	97	90
One localized Nodule	5	7	4	9	4	8
Multiple Localized Nodules	2	3	3	4	1	4
Bilateral Nodules	0	0	0	0	0	0
***P* value**	0.98		0.80		0.43	
**Breast Tenderness**						
*No Tenderness*	90	92	93	91	96	91
Low Tenderness	7	6	6	8	4	7
Medium Tenderness	5	4	3	3	2	4
High Tenderness	0	0	0	0	0	0
***P*-value**	0.59		0.40		0.31	
**Breast Discharge**						
No Discharge	88	90	91	89	91	93
One Duct Discharge	10	9	8	8	9	5
Multiple Ducts Discharge	4	3	3	5	2	4
Bilateral Discharge	0	0	0	0	0	0
***P*-value**	0.37		0.33		0.26	

Data are expressed as number. *P*-values refer to chi-square test or Fisher's exact test when appropriate

## Discussion

Our data show that daily intake of 50 mg isoflavone not only reduces the number and severity of hot flushes but also is safe for endometrial and breast tissue.

Many studies have shown positive effects of PEs on reducing the vasomotor symptoms of menopause (2, 15, 16), and their results are similar to the present study. However, many others have provided negative answers (17–19). A meta-analysis was performed including 15 high quality randomized clinical trials (RCTs) which investigated the effect of PEs on hot flushes and showed that this compound would significantly reduce the frequency of hot flushes in comparison to the placebo. In addition, there was no difference between PEs and placebo consumption in the occurrence of side effects. In two other review article and meta-analyses, researchers declared that studies on the effectiveness of PEs were so highly heterogeneous that they were unable to reach conclusive evidence (12, 20). About 43 RCTs were used with a total of 4364 participants to perform a meta-analysis of the literature about PEs use for alleviating menopausal vasomotor symptoms but found that very few studies were actually suitable for meta-analysis, therefore they were unable to definitely confirm the efficacy of PEs (12).

Although, there are many studies dedicated to investigating the effects of soy products on menopausal symptoms and subsequent quality of life in postmenopausal women, their results are controversial. The reasons for this disagreement can be categorized into 4 areas: I) different patients characteristics such as age range, duration of the menopausal age, the number and severity of hot flashes II) different study design in terms of sample size, duration of intervention, and different products of soy isoflavone consumed III) significant placebo effect in reducing hot flushes IV) individual differences in metabolism of isoflavone which depends on intestinal flora (21–23). Isoflavone has mixed estrogen agonist-antagonist properties (24). This effect has been made through two different mechanisms; I) Isoflavone selectively binds to β estrogen receptor, with less affinity and milder action than endogenous estrogen (25) II) they stimulate the synthesis of Sex Hormone Binding Globulin diminishing estradiol from the circulation (26).

Therefore, isoflavone would mildly affect the breast tissue. Clinical data on this subject is controversial: As many studies have provided evidence suggesting the protective effect of isoflavone against breast cancer (27, 28), they might induce proliferation of breast cancer cells (29). Isoflavone might interfere with selective estrogen receptor modulators such tamoxifen and aromatase inhibitors (30, 31). Most studies dedicated to evaluating the relation between isoflavone intake and breast risk used mammographic density as a biomarker for cancer risk but so far no association between serum PE levels and mammographic density has been detected (32, 33). Eight RCTs were studied which surveyed the effect of isoflavone-rich foods or supplements against placebo on breast density with a duration of at least 6 months intake in a systematic review and was showed that isoflavone intake does not alter breast density in postmenopausal women, but may cause a small increase in breast density in pre-menopausal women (31, 34).

However, we couldn’t find any significant differences in BE or TVS in our previous study with the same design (35) and the present study with more sample size confirmed our results.

Another caution about the use of isoflavone is due to its effect on the endometrium. One evidence with small participant number (27 patients) in 2002 reported no effect of isoflavone supplementation on endometrial histology (36). Further study with more sample size (376 patients) was investigated in randomized, double blind, placebo-controlled study over 5 yr and reported increased rates of endometrial hyperplasia. In their study, no cases of hyperplasia were observed in the placebo group, but 6 out of 154 women (3.8%) in the isoflavone taking group developed hyperplasia at the end of the follow-up period (37). Although 5 out of 6 of these cases were simple hyperplasia, one case of complex hyperplasia without atypia was seen in the entire study population. Considering the recent study in 2013, three-year intake of isoflavone supplementation had no effect on endometrial thickness or on rates of endometrial hyperplasia and cancer in postmenopausal women (38).

Since the high correlation between intra-observer and inter-observer measurements bias of endometrial thickness was proved (39), all endometrial thickness evaluations were done by the same radiologist, who has extensive gynecologic ultra-sound experience in this study. Our results were not manifested any significant changes in endometrial thickness during follow-up.

## Conclusion

This study is suggestive for advantageous effects of isoflavone on frequency and severity of hot flushes in perimenopausal women. We also demonstrated some clinical and ultrasonographic benign non-significant changes of the breast and endometrium induced by isoflavone. Therefore, future randomized-double-blinded placebo-controlled studies are needed to guarantee safe use of isoflavone in perimenopausal women especially those with the previous history of breast or endometrial cancers.

## Ethical considerations

Ethical issues (Including plagiarism, informed consent, misconduct, data fabrication and/or falsification, double publication and/or submission, redundancy, etc.) have been completely observed by the authors.
